# The Longitudinal Impact of Private Health Insurance on the Usage of Outpatient Services Among Aging Koreans

**DOI:** 10.1093/geroni/igaa057.345

**Published:** 2020-12-16

**Authors:** Narae Kim

**Affiliations:** University of Southern California, Los Angeles, California, United States

## Abstract

South Korea has a universal national health insurance system; however, its coverage is only about 65%. Consequently, many Koreans have supplemental private health insurance. This study aims to examine 1) how utilization of health care services and out-of-pocket expenditure of outpatient services varies by private health insurance status and 2) whether this relationship changes for these individuals over time. We analyze six waves of the Korean Longitudinal Study of Aging (KLoSA) from 2006 to 2016. Our primary outcomes of interest are the total number of outpatient visits and total out-of-pocket spending on outpatient services per year. Our independent variable of interest is private health insurance holding status. We use simple OLS regressions for each year to test if differences exist and change over time, controlling for age, sex, education, income, and various indicators of health conditions. The difference between those who never had private health insurance and those who always or sometimes had private health insurance becomes larger over time. When comparing total out-of-pocket expenditure, those who always had private health insurance have the lowest spending at the beginning of the study period (2006); however, their spending increases sharply by 2010 and remains higher than the other groups for the rest of the study period. Findings suggest that those who always had supplemental insurance use outpatient services more frequently and spend more out-of-pocket for services as a result. In addition, discrepancies become larger over time. As private health insurance holders age, the risk of higher utilization of outpatient services grows.

